# Novel strategy for ostial left anterior descending artery acute myocardial infarction: Combined treatment with directional coronary atherectomy followed by drug‐coated balloon angioplasty

**DOI:** 10.1002/ccr3.3659

**Published:** 2021-01-12

**Authors:** Hiroyuki Yamamoto, Takahiro Sawada, Tomofumi Takaya, Hiroya Kawai, Yoshinori Yasaka

**Affiliations:** ^1^ Division of Cardiovascular Medicine Department of Internal Medicine Hyogo Brain and Heart Center Himeji Japan; ^2^ Division of Cardiovascular Medicine Department of Exploratory and Advanced search in Cardiology Kobe University Graduate School of Medicine Kobe Japan

**Keywords:** acute myocardial infarction, bifurcation, directional coronary atherectomy, drug‐coated balloon, percutaneous coronary intervention

## Abstract

A 69‐year‐old female diagnosed with ostial left anterior descending artery acute myocardial infarction underwent percutaneous coronary intervention using combined directional coronary atherectomy followed by drug‐coated balloon angioplasty. This report highlights a novel management strategy with no permanent scaffold left in the coronary artery.

## Graphical abstract

Directional coronary atherectomy followed by drug‐coated balloon angioplasty may be a potentially effective therapeutic option without permanent scaffold in the coronary artery even in acute coronary syndrome.

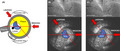

## 1 INTRODUCTION

A 69‐year‐old female diagnosed with ostial left anterior descending artery acute myocardial infarction underwent percutaneous coronary intervention using combined directional coronary atherectomy followed by drug‐coated balloon angioplasty. This report highlights a novel management strategy with no permanent scaffold left in the coronary artery.

Ostial left anterior descending artery (LAD) acute myocardial infarction (AMI) is associated with high morbidity and mortality owing to a broad ischemic area.^1^ Involvement of the left main trunk (LMT) bifurcation makes it challenging treatment‐wise; hence, several studies recommend stenting for complete plaque coverage from ostial LAD to LMT with crossover stenting.^2^, ^3^ However, LMT stenting may be unsuitable in some patients, including crossover stenting in younger patients and those with a high bleeding risk because of prolonged dual antiplatelet therapy (DAPT).

Drug‐coated balloon (DCB) is a safe and efficacious new device for stent‐less strategies requiring angioplasty in those with a high bleeding risk; this approach can be used in those with bifurcated lesions and even in patients with ST‐segment elevated AMI.^4^ However, optimal lesion preparation is necessary for treatments involving DCB angioplasty alone.^5^ Directional coronary atherectomy (DCA) is a unique technique to reduce coronary plaque in target lesions. Several studies have reported that DCA before stent implantation is effective and useful to avoid complex stenting for bifurcated lesions.^6^ Recently, an improved novel DCA catheter (Atherocut™, NIPRO) has been developed in Japan and was commercialized since 2015. A multicenter retrospective study showed that combination therapy with DCA and DCB could provide an option for stent‐less management of LMT bifurcated lesions in patients with stable coronary artery disease.^7^ However, the efficacy of the combination therapy with DCA and DCB in AMI remains unclear.

Herein, we describe a stent‐less treatment strategy in a patient with ostial LAD‐AMI using a DCB following DCA with serial changes in plaque morphology followed by use of intravascular ultrasound (IVUS). The patient agreed with this treatment before percutaneous coronary intervention (PCI) and provided informed consent for publication of the case.

## 2 CASE REPORT

**FIGURE 1:**
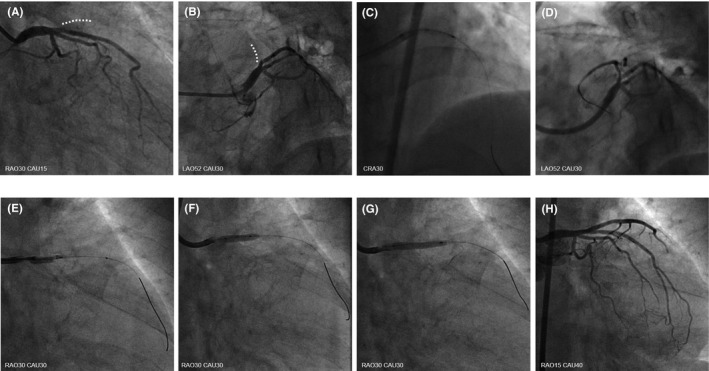
Angiogram during procedures. (A, B) Angiogram showing the occlusion at the ostium of left anterior descending artery, (C) thrombectomy, (D) angiogram after thrombectomy and angioplasty with 2.0‐mm balloon, (E) angiogram during directional coronary atherectomy, (F) angioplasty with a 3.5‐mm scoring balloon, (G) angioplasty with a drug‐coated balloon 3.5/20 mm, and (H) final angiogram

**FIGURE 2:**
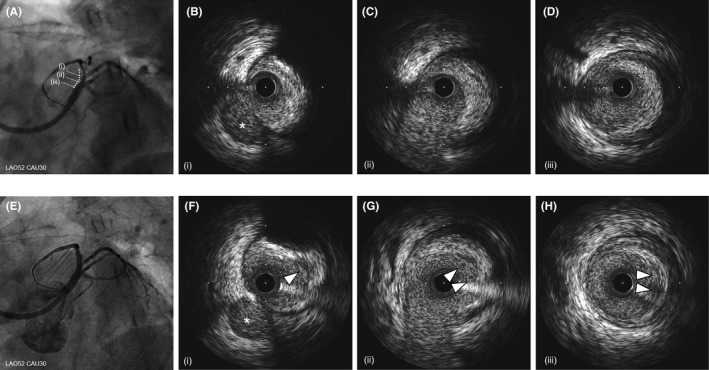
Angiogram and intravascular ultrasound findings before procedure and just after directional coronary atherectomy. (A) Angiogram after angioplasty with 2.0‐mm compliant balloon following thrombectomy, (B‐D) intravascular ultrasound (IVUS) images before directional coronary atherectomy (DCA); (B) at the culprit site, (C) at the left anterior descending artery (LAD) ostium, (D) at the left main trunk artery (LMT), (E) angiogram after DCA alone, (F‐H) IVUS images just after DCA; (F) at the culprit site, (G) at the LAD ostium, and (H) at the LMT (*; side branch, white arrow; atherectomy findings by DCA). Both lumen area and external elastic membrane area at culprit and LAD ostium sites were enlarged

**FIGURE 3:**
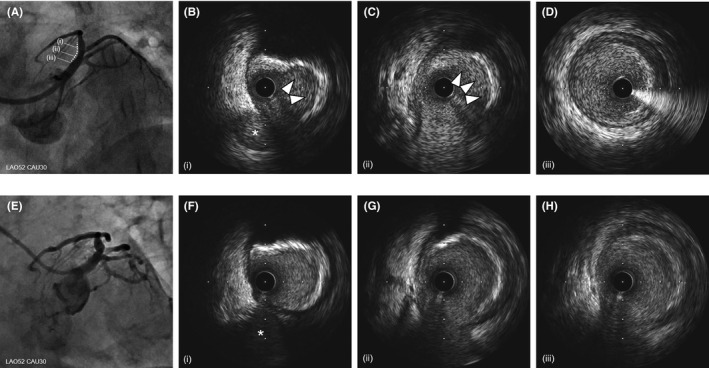
Angiogram and intravascular ultrasound findings at index percutaneous coronary intervention and the 9‐month follow‐up. (A) Angiogram after directional coronary atherectomy (DCA) and drug‐coated balloon (DCB) procedure, (B‐D) intravascular ultrasound (IVUS) images after DCA and DCB; (B) at the culprit site, (C) at the ostium of left anterior descending artery (LAD), (D) at the left main trunk artery (LMT), (E) angiogram at 9‐month follow‐up, (F‐H) IVUS images at 9‐month follow‐up; (F) at the culprit site, (G) at the LAD ostium, and (H) at the LMT (*; side branch, white arrow; atherectomy findings by DCA). Both late lumen and external elastic membrane enlargement were confirmed at the follow‐up

A 69‐year‐old woman previously treated for hypertension, dyslipidemia, and gastric ulcer in another hospital presented with sudden left‐sided chest pain radiating to the left arm. Electrocardiography showed ST‐segment elevation in the precordial leads, and an echocardiogram confirmed reduced left ventricular motion in the broad anterior wall. Hence, ST‐elevated AMI was suspected. She was hemodynamically stable (blood pressure was 135/89 mmHg, and pulse rate was 89‐/min), and she underwent an urgent coronary angiogram via the femoral artery because the radial artery was difficult to puncture due to narrowing or spasm. Urgent coronary angiography (CAG) using a 5‐Fr catheter confirmed occlusion of the ostial LAD (Figure 1A,B). Because our patient had a high bleeding risk, as assessed using the PRECISE‐DAPT score: advanced age and the presence of anemia, hypertension, and a previous gastric ulcer,^8^ crossover stenting to the LMT was undesirable because of prolonged DAPT duration. Therefore, we thought that DCA could reduce the plaque volume, and stent‐less PCI with DCB angioplasty would prevent LMT crossover stenting. Subsequently, emergent PCI was started using an 8‐Fr Launcher JCL 4.0 (Medtronic). An initial thrombectomy aspirated a little red thrombus, and successful recanalization was achieved 20 minutes after starting angiography (door‐to‐balloon time: 49 minutes) (Figure 1C). Pre‐dilatation with a 2.0‐mm compliant balloon was performed for achieving coronary flow improvement (Figure 1D). Then, IVUS (Opticross^TM^, Boston Scientific, MA, USA) revealed partially attenuated plaques extending from the LAD ostium to the LMT, as expected, which suggested the need for crossover stenting to the LMT rather than to the LAD; ostial stenting was also necessary for preventing coronary adverse events in the midterm postoperative period (Figure 2A‐D). Therefore, we decided to perform DCA and exchanged the guidewire to a 300‐cm ABYSS DCA support wire (NIPRO, Osaka, Japan). Subsequently, a DCA catheter (Atherocut™, L‐size, NIPRO, Osaka, Japan) was delivered to the culprit site (Figure 1E). After 4 attempts and 13 atherectomy cuts, plaque reduction was confirmed without the occurrence of the coronary slow flow phenomenon and under stable hemodynamic conditions (Figure 2E‐H); this was followed by additional ballooning with a 3.5‐mm scoring balloon (Lacrosse NSE, NIPRO, Osaka, Japan) (Figure 1F). IVUS showed no deep dissection, hematoma, or residual thrombus postdilatation with the 3.5‐mm scoring balloon (Figure 3B‐D), and we performed drug delivery to the culprit lesion with a 3.5/20‐mm DCB (SeQuent Please, paclitaxel‐coated balloon, B. Braun, Melsungen, Germany) (Figure 1G). Both lumen area (LA) and external elastic membrane area (EEM) were obviously enlarged at both culprit site and LAD ostium [the culprit site: LA: 2.8 mm^2^, 8.3 mm^2^, and 9.3 mm^2^; EEM: 16.7 mm^2^, 17.1 mm^2^, and 18.3 mm^2^, at the timing of before DCA, after DCA alone, and after DCA followed by DCB, respectively], [the LAD ostium: LA: 5.8 mm^2^, 10.3 mm^2^, and 12.1 mm^2^; EEM: 20.2 mm^2^, 21.9 mm^2^, and 22.2 mm^2^, at the timing of before DCA, after DCA alone, and after DCA followed by DCB, respectively]. The percent plaque area (%PA) markedly decreased after DCA followed by DCB angioplasty, from 83.0% to 49.1% at the culprit site and from 71.4% to 45.7% at the LAD ostium (Figure 1H and 3A‐D). Total procedure time, radiation time, radiation dose, and amount of contrast agent including CAG were 87 minutes, 58 minutes, 1.7 Gy, and 160 mL, respectively. Maximum creatine phosphokinase and creatine phosphokinase‐myocardial band levels were elevated at 1253 IU/L and 116 IU/L, respectively. The patient recovered well and was discharged on postadmission day 11 without any cardiac complications. The patient remained asymptomatic during the 9‐month follow‐up under 6 months of DAPT (acetylsalicylic acid 100 mg/day, prasugrel 3.75 mg/day) and only aspirin after 6 months according to Japanese guidelines, with the ability to stop DAPT if major bleeding occurred. A follow‐up angiogram at 9 months revealed no restenosis (Figure 3E), and IVUS revealed plaque regression over 9 months (Figure 3F‐H) (%PA: 47.6% vs 49.1%, follow‐up vs. index PCI) and enlargement of the LA and of the EEM of the culprit site and the LAD ostium (LA: 10.1 mm^2^ vs 9.3 mm^2^, EEM: 19.2 mm^2^ vs. 18.3 mm^2^, follow‐up vs. index PCI at the culprit site, respectively) (Figure 3B,F), (LA: 12.9 mm^2^ vs 12.1 mm^2^, EEM: 22.8 mm^2^ vs 22.2 mm^2^, follow‐up vs index PCI at LAD ostium, respectively) (Figure 3C,G).

## 3 DISCUSSION

In our patient, DCA was performed at the culprit site from the proximal part of the LAD to the LMT, followed by DCB. IVUS at 9‐month postprocedure showed both luminal and EEM enlargement, and good healing without the need of scaffolds in the culprit lesion. This novel intervention with DCA followed by the usage of a DCB avoids stent implantation even in AMI cases with LAD ostial lesions.

Currently, PCI with drug‐eluting stents for AMI is widely performed, but treatment for ostial LAD‐AMI is occasionally difficult because crossover stenting from the LAD to the LMT is often required for optimal results. Crossover stenting over the LMT bifurcation is associated with restenosis of the side branch in the midterm and prolonged DAPT duration as complications.^9^ In recent reports, stent‐less interventions with DCB were shown to be useful, and the advantage of luminal enlargement after the use of DCBs has also been reported.^4^, ^10^ A recent randomized study showed that the DCB strategy was noninferior to drug‐eluting stent implantation in AMI patients with lesion modification of residual stenosis of <50%.^5^ Therefore, DCB treatment without scaffolds in the culprit site could be feasible in patients with ostial LAD‐AMI. However, the use of a DCB alone for LAD ostial lesions poses challenges. Without successful lesion preparation, bailout stenting may be required for extensive dissection and hematoma, and residual plaque burden after balloon angioplasty might significantly impact clinical outcomes postprocedure. Previous reports suggested that DCA is useful and effective for treating LMT bifurcation lesions, even in the plain old balloon era, because DCA avoided adverse effects in side branches, including carina and plaque shift after ballooning, despite a higher restenosis rate.^11^ We believe that the adjuvant effects of DCB angioplasty even in AMI patients who have undergone DCA would improve subacute restenosis rate by reducing neointimal proliferation. Most AMI cases are caused by plaque rupture and erosion, and large amounts of lipid plaques and thrombi exist in the culprit lesions. Although the DCA procedure might have a potential complication of slow flow phenomenon, DCA prior to optimal lesion modification with adequate balloon angioplasty might be suitable for reducing balloon‐related coronary slow flow, because of resection of residual vulnerable plaques directly from the coronary artery.

Chronic late lumen enlargement phenomena after only DCB angioplasty for de novo coronary artery disease have good outcomes.^10^, ^12^ In our patient, similarly, late lumen enlargement was noted in the culprit site and EEM area enlargement and plaque regression were noted on serial IVUS examination, with good long‐term angiographic findings, although late lumen enlargement in AMI cases might be affected by antiplatelet medication disturbing residual small thrombi. However, a previous study which evaluated serial changes on IVUS after DCA without the use of a DCB demonstrated a decreased EEM area and increased plaque area, causing restenosis.^13^ This discrepancy in serial IVUS findings was intriguing, and it may be attributed to the compatibility of the combined DCA and DCB treatment.

**FIGURE 4:**
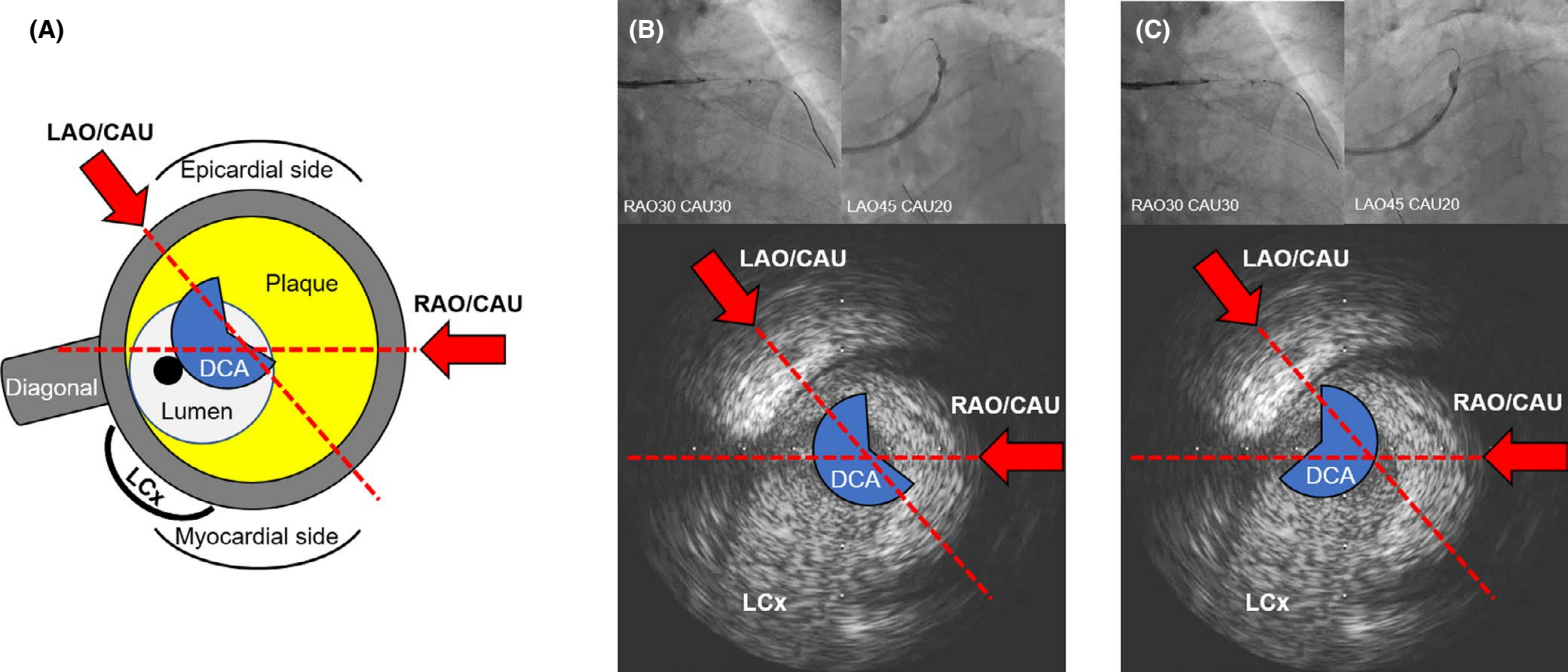
The role of bidirectional angiography and intravascular ultrasound during directional coronary atherectomy. (A) The schema shows an intravascular ultrasound (IVUS) cross‐sectional image of the left anterior descending artery. (B, C) When the angiogram from the right anterior oblique (RAO)/caudal (CAU) view suggests the housing is directed toward the plaque, the angiogram should be checked from another view. Angiogram from the left anterior oblique (LAO)/CAU view shows two patterns; (B) the housing of DCA adequately faces the direction of the target plaque, (C) the housing of DCA faces the opposite direction to the target plaque; evaluation with bidirectional angiogram is useful to confirm adequate direction of the DCA housing.

There are some key points to keep in mind with DCA procedure in AMI patients. Even in hemodynamically stable patients, the first DCA attempt often requires a special attention because a DCA catheter for large plaque volume might lead to coronary flow disturbance. However, in the subsequent attempt, flow disturbance is less likely to occur. The duration of temporary coronary flow disorder caused by DCA procedure should be as short as possible. Temporal use of vasopressor agents could stabilize the patient's hemodynamics and make the procedure easier to perform. In addition, this strategy has several problems. First, DCA is generally a complex procedure performed by experts; however, the novel DCA catheter is easy to use under the evaluation by IVUS, which is useful to detect plaque distribution for the adequate and sufficient debulking. We should check the direction of the DCA catheter by bidirectional angiography in order to avoid catastrophic complications, because this novel DCA catheter sometimes rotates to the opposite side due to a whip motion caused by residual torque force just after previous DCA procedure (Figure 4). Second, DCA is incompatible with the following: (a) patients with severe calcified lesions, (b) patients with poor renal function due to the possible requirement of more contrast volume than that used in conventional procedures, and (c) patients with hemodynamic instability under cardiac support devices. Third, coronary flow limitation in the left circumflex artery may occur and cause a catastrophic cardiac event. Therefore, this procedure may be unsuitable, especially in cases with dominant left coronary artery with a small right coronary artery. Fourth, the effectiveness of DCA for mainly thrombotic lesions remains unclear. Excimer laser coronary atherectomy (ELCA) is another option for AMI; however, the maximum available size of ELCA is 2.0 mm. DCA, which has adaptation for vessel size more than 3.0‐mm, would be suitable in the ostial LAD lesion rather than ELCA in terms of plaque debulking. However, ELCA and thrombus aspiration prior to DCA procedure might be effective in AMI cases with concerns of distal embolism, as was in our case. After thrombus reduction, DCA could be safely performed and be effective in the reduction of residual culprit plaque. Despite these limitations, we believe that angioplasty with the use of a DCB following DCA for residual plaques is effective and becomes one of the strategies as an alternative to LMT crossover stenting in high bleeding risk patients who can tolerate hemodynamic instability. Further observational studies or randomized clinical trials should address whether DCA followed by the DCB strategy is effective in terms of future cardiovascular events including hemorrhage complications compared with conventional strategy.

## 4 CONCLUSION

We report a stent‐less management for a high‐risk patient with ostial LAD‐AMI, wherein a DCB angioplasty following DCA avoided stenting and restenosis in the midterm. This combination of DCA and DCB treatments for ostial LAD‐AMI might be feasible for avoiding crossover LMT stent deployment in patients with a high bleeding risk. Further studies are warranted to investigate the efficacy of the use of DCB after DCA in patients with AMI.

## CONFLICT OF INTEREST

None declared.

## AUTHOR CONTRIBUTIONS

HY and TS: contributed to developing this stent‐less management and drafting the manuscript. TT, HK and YY: contributed to engaging technical support and supervised the study.

## ETHICAL APPROVAL

This manuscript has not been published elsewhere, and this treatment strategy has been approved by the appropriate ethics review board.

## Data Availability

The data of this case are available from the corresponding author upon reasonable request.

## References

[1] Brener SJ , Witzenbichler B , Maehara A , et al. Infarct size and mortality in patients with proximal versus mid left anterior descending artery occlusion: the intracoronary abciximab and aspiration thrombectomy in patients with large anterior myocardial infarction (INFUSE‐AMI) trial. Am Heart J. 2013;166:64‐70. 23816023 10.1016/j.ahj.2013.03.029

[2] Yamamoto K , Sakakura K , Akashi K , et al. Clinical outcomes of left main crossover stenting for ostial left anterior descending artery acute myocardial infarction. Heart Vessels. 2018;33:33‐40. 28776068 10.1007/s00380-017-1033-0

[3] Rigatelli G , Zuin M , Baracca E , et al. Long‐term clinical outcomes of isolated ostial left anterior descending disease treatment: ostial stenting versus left main cross‐over stenting. Cardiovasc Revasc Med. 2019;20:1058‐1062. 30797760 10.1016/j.carrev.2019.01.030

[4] Jeger RV , Eccleshall S , Wan Ahmad WA , et al. Drug‐coated balloons for coronary artery disease: third report of the international DCB consensus group. JACC Cardiovasc Interv. 2020;13:1391‐1402. 32473887 10.1016/j.jcin.2020.02.043

[5] Vos NS , Fagel ND , Amoroso G , et al. Paclitaxel‐coated balloon angioplasty versus drug‐eluting stent in acute myocardial infarction: the revelation randomized trial. JACC Cardiovasc Interv. 2019;12:1691‐1699. 31126887 10.1016/j.jcin.2019.04.016

[6] Tsuchikane E , Aizawa T , Tamai H , et al. Pre‐drug‐eluting stent debulking of bifurcated coronary lesions. J Am Coll Cardiol. 2007;50:1941‐1945. 17996557 10.1016/j.jacc.2007.07.066

[7] Kitani S , Igarashi Y , Tsuchikane E , et al. Efficacy of drug‐coated balloon angioplasty after directional coronary atherectomy for coronary bifurcation lesions (DCA/DCB registry). Catheter Cardiovasc Interv. 2020. 10.1002/ccd.29185 32776689

[8] Costa F , van Klaveren D , James S , et al. Derivation and validation of the predicting bleeding complications in patients undergoing stent implantation and subsequent dual antiplatelet therapy (PRECISE‐DAPT) score: a pooled analysis of individual‐patient datasets from clinical trials. Lancet. 2017;389:1025‐1034. 28290994 10.1016/S0140-6736(17)30397-5

[9] D'Ascenzo F , Barbero U , Abdirashid M , et al. Incidence of adverse events at 3 months versus at 12 months after dual antiplatelet therapy cessation in patients treated with thin stents with unprotected left main or coronary bifurcations. Am J Cardiol. 2020;125:491‐499. 31889527 10.1016/j.amjcard.2019.10.058

[10] Kleber FX , Schulz A , Waliszewski M , et al. Local paclitaxel induces late lumen enlargement in coronary arteries after balloon angioplasty. Clin Res Cardiol. 2015;104:217‐225. 25349065 10.1007/s00392-014-0775-2

[11] Tanaka N , Terashima M , Kinoshita Y , et al. Unprotected left main coronary artery bifurcation stenosis: impact of plaque debulking prior to single sirolimus‐eluting stent implantation. J Invasive Cardiol. 2008;20:505‐510. 18829993

[12] Piraino D , Buccheri D , Cortese B . Paclitaxel‐coated balloon exerts late vessel healing and enlargement: a documented phenomenon with optical coherence tomography analysis. Int J Cardiol. 2016;15:551‐552. 10.1016/j.ijcard.2015.10.165 26569361

[13] Lansky AJ , Mintz GS , Popma JJ , et al. Remodeling after directional coronary atherectomy (with and without adjunct percutaneous transluminal coronary angioplasty): a serial angiographic and intravascular ultrasound analysis from the optimal atherectomy restenosis study. J Am Coll Cardiol. 1998;32:329‐337. 9708457 10.1016/s0735-1097(98)00245-9

